# Hierarchical Mo_2_C@CNT Hybrid Structure Formation for the Improved Lithium-Ion Battery Storage Performance

**DOI:** 10.3390/nano11092195

**Published:** 2021-08-26

**Authors:** Sajjad Hussain, Shoaib Muhammad, Muhammad Faizan, Kyung-Wan Nam, Hyun-Seok Kim, Dhanasekaran Vikraman, Jongwan Jung

**Affiliations:** 1 Hybrid Materials Center (HMC), Sejong University, Seoul 05006, Korea; shussainawan@gmail.com; 2 Department of Nanotechnology and Advanced Materials Engineering, Sejong University, Seoul 05006, Korea; 3 Department of Energy Science, Sungkyunkwan University, Suwon 16419, Korea; mshoaibce@gmail.com; 4 Department of Energy & Materials Engineering, Dongguk University-Seoul, Seoul 04620, Korea; faizijaff@gmail.com (M.F.); knam@dongguk.edu (K.-W.N.); 5 Department of Materials Engineering, NED University of Engineering and Technology, Karachi 75270, Pakistan; 6 Division of Electronics and Electrical Engineering, Dongguk University-Seoul, Seoul 04620, Korea; hyunseokk@dongguk.edu

**Keywords:** hybrid, Mo_2_C, Mo_2_C@CNT, TMCs, CNT, LIBs

## Abstract

2-D transition metal carbides (TMCs)-based anode materials offer competitive performance in lithium-ion batteries (LIBs) owing to its excellent conductivity; cheaper, flexible uses; and superior mechanical stability. However, the electrochemical energy storage of TMCs is still the major obstacle due to their modest capacity and the trends of restacking/aggregation. In this report, the Mo_2_C nanosheets were attached on conductive CNT network to form a hierarchical 2D hybrid structure, which not only alleviated the aggregation of the Mo_2_C nanoparticle and facilitated the rapid transference of ion/electron, but also adapted effectually to the hefty volume expansion of Mo_2_C nanosheets and prevented restacking/collapse of Mo_2_C structure. Benefitting from the layered Mo_2_@CNT hybrid structure, the charge/discharge profile produced a 200 mAh g^−1^ discharge-specific capacity (second cycle) and 132 mAh g^−1^ reversible-discharge discharge-specific capacity (after 100 cycles) at 50 mA g^−1^ current density, with high-speed competency and superior cycle stability. The improved storage kinetics for Mo_2_@CNT hybrid structure are credited to the creation of numerous active catalytic facets and association reaction between the CNT and Mo_2_C, promoting the efficient electron transfer and enhancing the cycling stability.

## 1. Introduction

In the last few years, reusable Li-ion batteries (LIBs) have played a prominent part in energy storage for various devices, such as wearable/portable consumer electronics, implantable devices, vehicles and smart power grids, mobile phones, laptops, and so forth, due to its high energy density, being smaller/buoyant, and having excellent rate capability/cycling stability [1,2]. Graphite is commonly utilized as an anode in industrial LIBs, but it still struggles to reach the high capacities and energy densities necessary for electronic gadgets because of its low theoretical specific capacity of 372 mA h g^−1^, which prominently confines its societal development [3,4,5]. Therefore, great endeavors have been made to seek the design of advanced high capacity electrode materials with swift intercalation performance, cheap cost, and superior robustness for next generation LIBs [6,7].

Two-dimensional transition metal carbides (TMCs) and nitrides are fascinating active electrode systems because of their beneficial physical behaviors of high melting points and electrical conductivity, as well as robust chemical stability [8,9]. Among the various investigated TMC materials, molybdenum carbide (Mo_2_C) has received particular attention as an emerging anode electrode because of its enormous theoretical capacity, which far exceeds that of amorphous carbon, and outstanding electrical conductivity, low cost availability, excellent electrochemical behavior, and identical electronic configuration of Pt group metals [10,11,12,13,14]. Theoretical calculations also validated that Mo_2_C has a minimal Li^+^ scattering fence of 0.035 eV, which implies their proficiency for Li^+^ swift storage and discharge and greatly widens their application [12,14]. However, experimental results showed poor cyclability and rapidly declining capacity of bulk Mo_2_C for LIBs [13,15]. Furthermore, the inevitable accretion of Mo_2_C particles during the electrochemical reaction leads to less exposed active sites or reduced electrons/protons transport to the electrode surface from the electrolytes, thereby decreasing the intercalation/de-intercalation for Li-ion transport as well as deteriorating the device enactment. To overcome these aspects, many approaches have been widely practiced such as designing the nanocomposites to attenuate nanocrystal aggregation, providing a large active surface to interact with electrolytes, enhancing the charge-transfer process and refining the structural durability [16,17]. To enhance the electrochemical behavior of Mo_2_C-based electrode systems and induce its real potential towards Li^+^ storage, a variety of hybrid materials were rationally designed with different materials to tune the geometric shapes and morphologies at the nanoscale level [15,18]. The hybrid materials formation would improve the surface between the electrolyte and electrode and reduce the diffusion pathways of lithium ions/electrons, thereby ensuring maximum capacity and better frequency performance [15]. With all these significant advantages, porous 3D CNTs network has also been extensively employed as an anode material in Li-ion/Na-ion batteries due to its 1D structure with a long span to diameter proportion, high conductivity, specific weight, and higher mechanical properties, and is associated with high absorbency and specific area, which are credited for easy facilitation of rapid transportation of electrons/ions, thus leading to good cycling behavior [19,20]. However, these CNT-based electrodes still have delivered relatively moderate capacities. Until now, the use of CNT-based materials such as high-capacity electrode materials for LIBs such as peapod-like Co_3_O_4_@CNT [21], self-assembled MoS_x_/CNT nanocomposites [22], graphene oxide/graphite/CNT composite [23], single-walled carbon nanotube/SnO_2_ (SWCNT/SnO_2_) [24], Mo_2_C nanoparticles/graphene(GR) hybrid [15], mesoporous Mo_2_C–C hybrid nanospheres [13], N-doped porous CNT/Mo_2_C [25], W_2_C/WS_2_ alloy nanoflowers [26], MoS_2_ nanosheet/CNT Composite [27], N-doped graphene/MoS_2_/N-doped graphene heterostructure [28], MoS_2_ shells-supported carbon spheres [29], and eutectoid WxC embedded WS_2_ nanosheets [9] have been reported. 

Herein, Mo_2_C@CNT composites are proposed as anode material to increase inner porosity, reduce dense stacking, and bring about strong intimate contact and high ion accessibility, which could further lead to better speed competency, high revocable capacity, and cycle steadiness. Benefiting from the high conducting nature of Mo_2_C and CNTs, Mo_2_C@CNT exposed the excellent cyclability with a rescindable 132 mAh g^−1^ discharge capacity at 50 mA g^−1^ current density after 100 cycles. Our results indicate that active material of Mo_2_C nanoparticles wrapping/attaching into porous CNT nets is an efficient process to enhance the reversible capacity and avoid accretion of these nanoparticles in the repetitive battery cycling process.

## 2. Materials and Methods 

### 2.1. Synthetic of Mo_2_C@CNT Hybrids

The chemical reduction approach was adopted for synthesizing the Mo_2_C from commercial samples as reported in our previous study [30]. The viable CNT was purchased from Hanwha Nanotech (multiwalled CNT, CM-95). The reduced Mo_2_C nanoparticles and CNTs were well blended with 1:1 weight ratio in ethanol (50 mL) for the Mo_2_C@CNT hybrid preparation, and the solution was extensively sonicated using an ultrasonic bath. The resulting black mixture was then vigorously agitated at 80 °C for 12 h until ethanol was drained. Further, the residues were centrifuged for 15 min at 10,000× *g* rpm in ethanol and deionized (DI) water, and dried at 80 °C in a furnace overnight. The obtained powder samples were annealed in CH_4_ environment with a mixture of H_2_/Ar gas for 3 h at 800 °C. The many characterizations were analyzed to confirm the properties of nanostructures and their instrument details are given in the supporting information.

### 2.2. Electrochemical Measurements

The poly (vinylidene fluoride) binder ~10 wt%, carbon black ~10 wt%, and active material ~80 wt%, with N-methyl-2-pyrrolidone (NMP), were employed to arrange uniform slurry for the anode fabrication. As-prepared slurry was further coated on a 25-mm-thick Cu foil and dehydrated in vacuum for 24 h at 60 °C to use as anode. The coin cells were fabricated using Celgard 2500 membrane (separator), lithium (Li) foil (counter electrode), and active material-loaded Cu foil (anode) along with the LiPF_6_ (1 M) in dimethyl carbonate/ethylene carbonate (1:1) electrolyte by glove box in an Ar atmosphere. The electrochemical studies were accomplished using a battery cycler (Wonatech, WBS3000) at ambient temperature. Electrochemical impedance spectroscopy (EIS) measurement was operated at a frequency of 100 kHz–0.01 Hz using the electrochemical unit PGSTAT 302 (Metrohm Autolab B.V.). Cyclic voltammetry (CV) studies were tested by electrochemical workstation (VMP3, Bio-logic, Claix, France). All the electrochemical results were confirmed by the triplicated measurements. 

## 3. Results and Discussion

### Materials Characteristics

**Figure 1:**
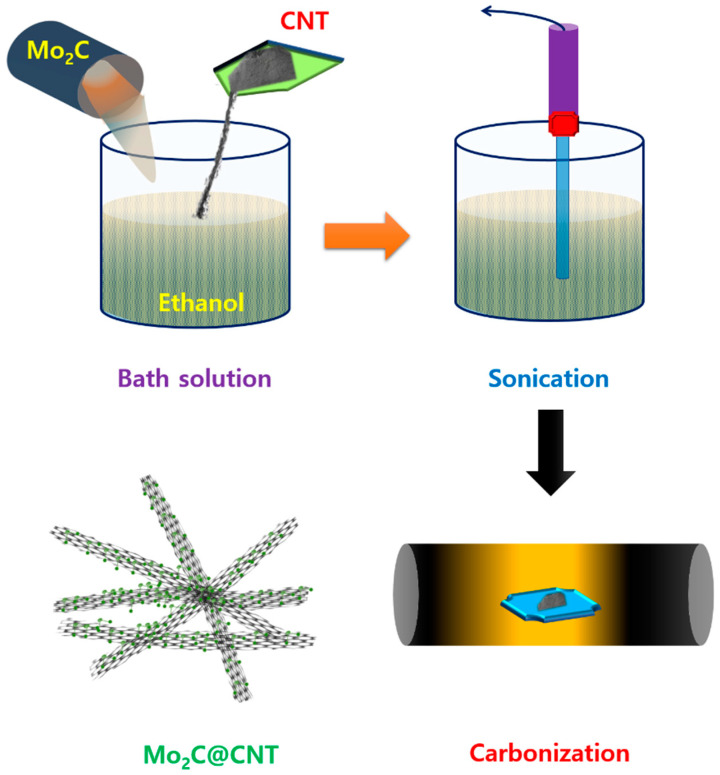
Graphic presentation for the synthesis of Mo_2_C@CNTs hybrid.

**Figure 2:**
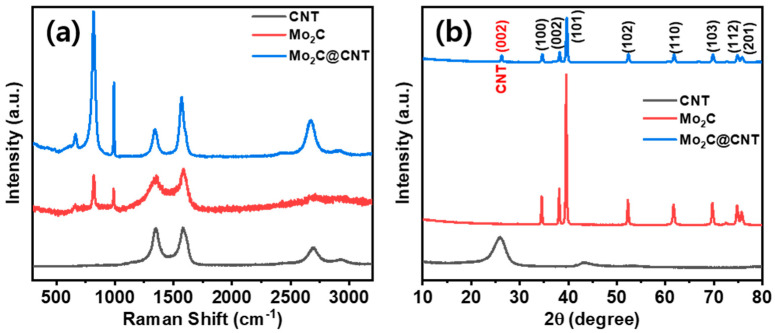
(**a**) XRD using Cu-Kα radiation (0.154 nm) and (**b**) Raman scattering using Ar laser source (512 nm) spectra of Mo_2_C, CNTs, and Mo_2_C-CNTs.

Figure 1 shows a scheme of the Mo_2_C@CNT preparation process by ultrasonic reaction followed by the carbonization process. Raman spectra were developed to study the formation of CNT, Mo_2_C, and Mo_2_C@CNT nanocomposites. Figure 2a displays the Raman profiles of CNT, Mo_2_C, and Mo_2_C@CNT nanocomposites. The distinctive characteristic peaks of D-band (1342 cm^−1^) and G-band (1573 cm^−1^) are perceived from the CNT. Moreover, the characteristic peaks of 2D-band and G+D-band emerged at 2702 cm^−1^ and 2928 cm^−1^, respectively [31]. Mo_2_C generates the distinguishing peaks at 990 cm^−1^, 818 cm^−1^, and 661 cm^−1^, ascribed to β-Mo_2_C. After the hybridization of Mo_2_C@CNT, the slight shifts are observed in the peak position due to the interchanging of atoms across the interface, realizing the strong coupling between Mo_2_C and CNT [8]. The strong intimate contact of hybrid is beneficial for the cycling stability.

Moreover, to explore the structural properties of CNT, Mo_2_C, and Mo_2_C@CNT, X-ray diffraction (XRD) analyses were done. Figure 2b illustrates the XRD patterns of CNT, Mo_2_C, and Mo_2_C@CNT nanostructures. The reduced Mo_2_C generates reflections at 2θ = 34.6, 38.0, 39.4, 52.3, 61.7, 69.9, 74.7, and 75.8° matching planes of (100), (002), (101), (102), (110), (103), (112), and (201) to confirm β-Mo_2_C (JCPDS:35-0787). The strong diffraction line is at 2θ = 25.9° along with a wide pathetic peak at 43.2° corresponding to the (002) and (101) reflections, respectively, of pristine CNT sample. For the Mo_2_C@CNT, integrated network complying the diffraction planes at 2θ = (100), (002), (101), (102), (110), (103), (112), and (201) lattices of Mo_2_C are exposed at 34.5°, 38.3°, 39.7°, 52.5°, 61.8°, 69.7°, 74.9°, and 75.9°, respectively (JCPDS-893014). Moreover, the narrowed width (002) plane of CNT originated from Mo_2_C@CNT, indicating the development of interacted hybridized material.

**Figure 3:**
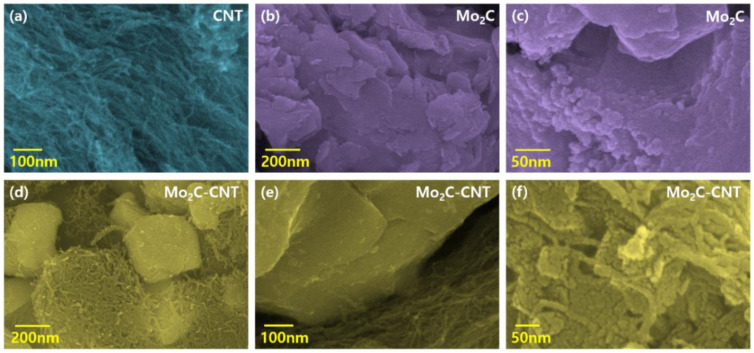
FESEM imagery of (**a**) CNT, (**b**,**c**) Mo_2_C, and (**d**–**f**) Mo_2_C@CNThybrid.

**Figure 4:**
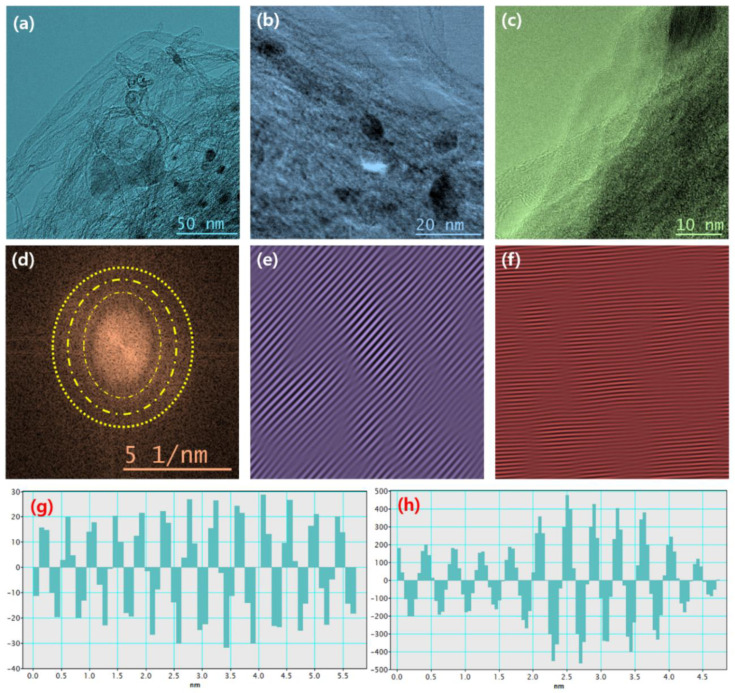
HRTEM of Mo_2_C@CNT hybrid: (**a**–**c**) Typical HRTEM images with different magnifications; (**d**) FFT pattern; (**e**) iFFT pattern and (**g**) phase profile for Mo_2_C lattices; and (**f**) iFFT pattern and (**h**) phase profile for CNT lattices.

The field emission scanning electron microscopy (FESEM) measurement was performed to examine the morphological behavior of CNT, Mo_2_C, and Mo_2_C@CNT hybrids. Figure 3a shows a CNT high-resolution FESEM image, which demonstrates the existence of well-structured multi-walled nanotubes. The combined array of agglomerated nanograins is exhibited for Mo_2_C as shown in Figure 3b. Further, high resolution image (Figure 3c) clearly proves the formation of spherical-shaped nanograins. For the hybrid structure, FESEM images clarified that the agglomerated Mo_2_C grains are well interconnected with nanotubes (Figure 3d–f). Further, high resolution imageries visibly establish the accumulated larger-size Mo_2_C grains bounded by CNT. The intersection between the CNT and Mo_2_C with the decoration of spherical nanograins is evidenced in the high resolution FESEM micrograph (Figure 3f). Figure S1 contains the elemental mapping images to confirm the equally distributed elements in the hybrid. Figure 4a–d shows the FESEM images for Mo_2_C@CNT. Moreover, to understand the hybrid nanostructure formation, high-resolution transmission electron microscopy (HRTEM) studies were done for Mo_2_C@CNT. Figure 4a of low magnification HRTEM realizes the CNT-embraced Mo_2_C nanoparticles for the Mo_2_C@CNT. The selective region in HRTEM produces, as shown in Figure 4b, the dusky nature of Mo_2_C grains along with the white shaded contour by CNT. Further, the high resolution HRTEM shows the different interfaced zones of the hybrid nanostructure (Figure 4c). The extracted FFT pattern, as shown in the Figure 4d, is indicated by the Moire ring structure due to their well-defined lattice planes, which are emphasized with dotted lines [32]. The extracted inverse FFT pattern reveals the unique lattice direction of Mo_2_C and CNT, respectively, as exposed in Figure 4e,f. Figure 4g explores the phase profile form of Figure 4e and their space of 0.47 nm correlates to (001) Mo_2_C direction. Figure 4h displays the phase pattern of Figure 4f iFFT with 0.34 nm owing to the (002) CNT lattice direction.

**Figure 5:**
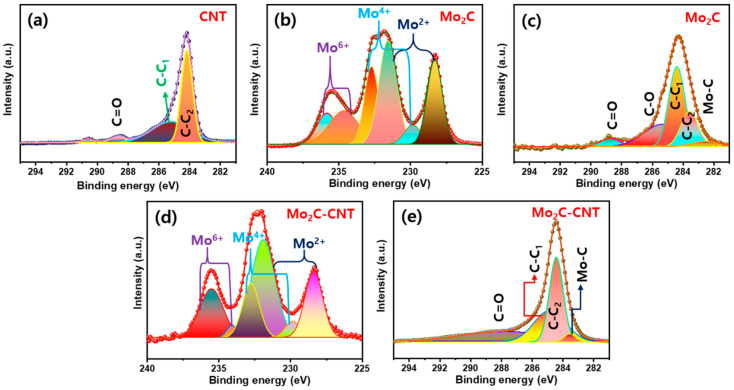
XPS results of (**a**) C 1s for CNT; (**b**) Mo 3d and (**c**) C 1s for Mo_2_C; and (**d**) Mo 3d and (**e**) C 1s for Mo_2_C@CNT.

The chemical compositions of Mo_2_C, CNT, and Mo_2_C@CNT nanostructures were studied via X-ray photoelectron spectroscopy (XPS). Figure 5a displays the C 1s region XPS outline, which explores the C=O (288.5 eV), sp^2^ C-C (284.2 eV), and sp^3^ C–C (285.1 eV). For the Mo_2_C, in the Mo 3d region (Figure 5b) originates the Mo^4+^ (229.8 and 232.7 eV), Mo^2+^ (228.3 and 231.5 eV), and Mo^6+^ (234.6 and 235.8 eV) pairs of 3d_5/2_ and 3d_3/2_, respectively, due to the typical Mo_2_C [33,34]. Moreover, C 1s region (Figure 5c) creates the broad carbon-related characteristic peak of sp^2^ C–C, sp^3^ C–C, C–O, and C=O along with the Mo-C band (282.5 eV) [8,35]. In the case of Mo_2_C@CNT, Mo 3d and C 1s regions obviously produce the characteristic peaks as displayed in Figure 5d,e, respectively [30]. Figure S2 shows the survey spectrum of Mo_2_C@CNT nanostructures.

**Figure 6:**
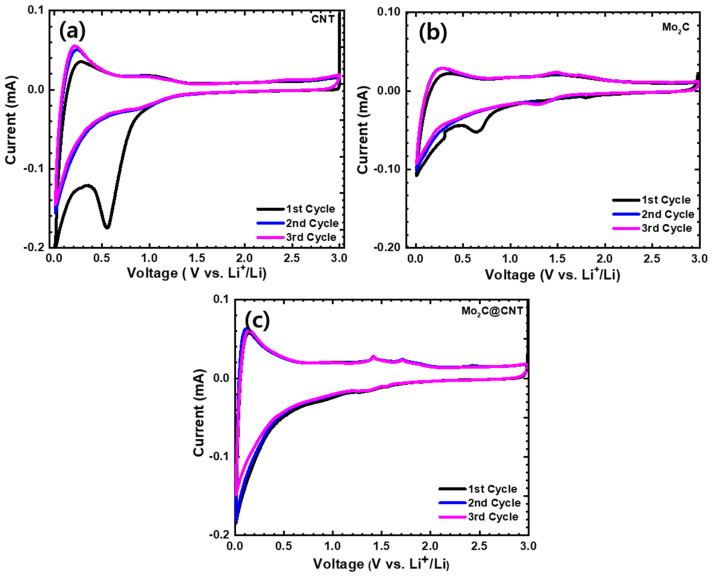
Cyclic voltammograms at 0.1 mV·s^−1^ in the range of 0.01–3.0 V for (**a**) CNT, (**b**) Mo_2_C, and (**c**) Mo_2_C@CNT hybrid anodes.

To illustrate the electrochemical behavior, CNT, Mo_2_C, and Mo_2_C@CNT hybrid nanostructures were prepared as the anode material for LIBs. The CV studies were carried out at 0.01–3.0 V (vs. Li/Li^+^) voltage range at 0.1 mV s^−1^ sweep rate. Figure 6 displays the first three cycles of CV for the CNT, Mo_2_C, and Mo_2_C@CNT hybrid anodes. The fabricated CNT anode (Figure 6a) produces a sharp irreversible peak at 0.5 V in the initial cathodic scan, which associates to the realization of solid-electrolyte interphase (SEI) film [36]. At the second and third cycles, the identical CV profiles are perceived for the CNT anode. Figure 6b displays the first three cycle CVs of Mo_2_C anode. In the case of Mo_2_C, a reduction peak appears at about 0.63 V in the initial cycling and vanishes in consequent cycles, with an irreparable peak associated to disintegration of the electrolyte and SEI foundation. Two small apparent redox couples are exposed from the second and third cycles at approximately 1.46/1.32 V during the lithiation/extraction kinetics, which is related to the exchange process of Mo_2_C with Li^+^ and the oxidation kinetic of Li^+^ release from Li_x_C, respectively. Mo_2_C indicates a reversible conversion reaction or alloying reaction between the Li^+^ and Mo_2_C (e.g., xLi^+^ + Mo_2_C + xe^−^ → Mo + Li_x_C) [13,15]. These results agree with earlier Mo_2_C literature [37]. Figure 6c shows the first three cycle CVs of the Mo_2_C@CNT hybrid anode. The highly reversible CV profiles are observed for the hybrid anode along with redox couples at 1.48/1.35 V during the lithiation/extraction kinetics.

**Figure 7:**
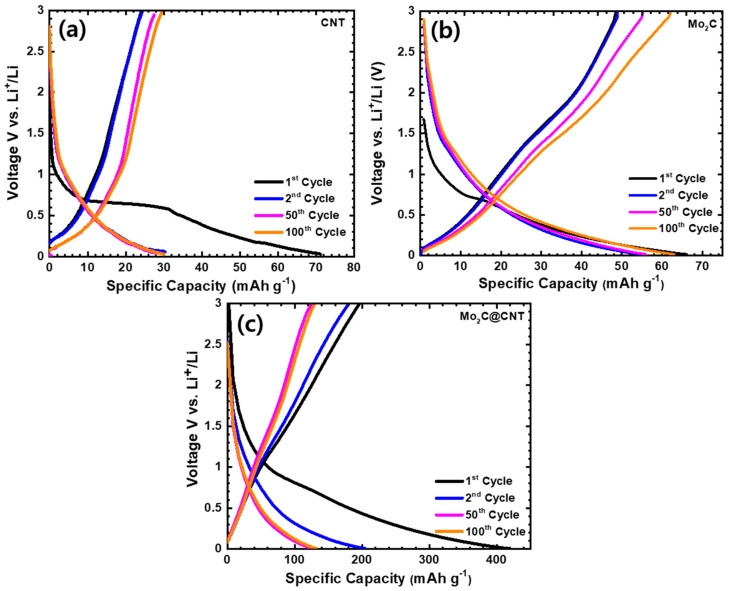
Charge-discharge at 50 mA g^−1^ in the range of 0.01–3.0 V for (**a**) CNT, (**b**) Mo_2_C, and (**c**) Mo_2_C@CNT hybrid anodes at the 1st, 2nd, 50th, and 100th cycle for lithium-ion battery.

The distinctive discharge/charge curves of LIBs using CNT, Mo_2_C, and Mo_2_C@CNT anode over the 0.01–3.0 V voltage window were performed at 50 mA g^−1^ current density. Figure 7a–c shows the charge/discharge profiles of CNT, Mo_2_C, and Mo_2_C@CNT anodes. As expected, electrochemically inactive material of 2D Mo_2_C and CNT material shows inferior capacity. The observed discharge capacities are at 66 and 71 mAh g^−1^, respectively, for the CNT and Mo_2_C anode. The Mo_2_C@CNT anode provides enriched capacity due to the interaction effect of both materials. Moreover, 416 mAh g^−1^ of the first discharge capacity is perceived for the hybrid anode and then a loss of 49% of capacity after the second discharge; this fading is due to the crosswise process and realization of SEI deposit. Subsequently, the capacity maintains approximately 132 mAh g^−1^ after 100 cycles for the hybrid anode as observed in the Figure 7c. The observed values are more comparable to most 2D materials-based anodes such as LiMn_2_O_4_/graphene (137 and 101 mAh g^−1^ at 1 and 100 C rate.) [38], MWCNTs-modified LiVPO_4_F (132.4 mAh g^−1^ at 0.5 C) [39], MoS_2_/C (400 mAh g^−1^ at 100 mAg^−1^ after 50 cycles) [40], nano-porous carbon (495 mAh g^−1^ after 100 cycles at 0.2 °C) [41], WS_2_/rGO composite (431.2 mAh g^−1^, at 0.1 A g^−1^ after 100 cycles) [42], C@MoS_2_@PPy (294 mAh g^−1^ at 5 A g^−1^ after 500 cycles), and flower like TiO_2_-B nanoparticles wrapped by graphene nanosheets (171 mA h g^−1^ at 5 C for 100 cycles) [43]. The better retention behavior can be observed by the synergistic interaction between CNT and Mo_2_C, which unremittingly shows the stable electrical and ionic conductivity.

In addition, an EIS measurement was performed for the CNT, Mo_2_C, and Mo_2_C@CNT anodes to further analyze the electrochemical kinetics. Figure S3 shows Nyquist plots and a fitted electrical circuit for the CNT, Mo_2_C, and Mo_2_C@CNT anodes. It is found that the R_ct_ value of the CNT, Mo_2_C, and hybrid are at 879.5 ± 1.2 Ω, 885.5 ± 0.8 Ω, and 824.6 ± 0.9 Ω, respectively. The hybrid anode shows better conductivity than the single components. This conductivity is benefitted through the conductive matrix of CNT as well as reduced particle size of Mo_2_C, which enable the fast movement of Li^+^ ions in the composite electrode. The stable SEI layer also favors the fast ion transfer due to which the composite shows stable cycling and appreciable capacity at high current density. 

**Figure 8:**
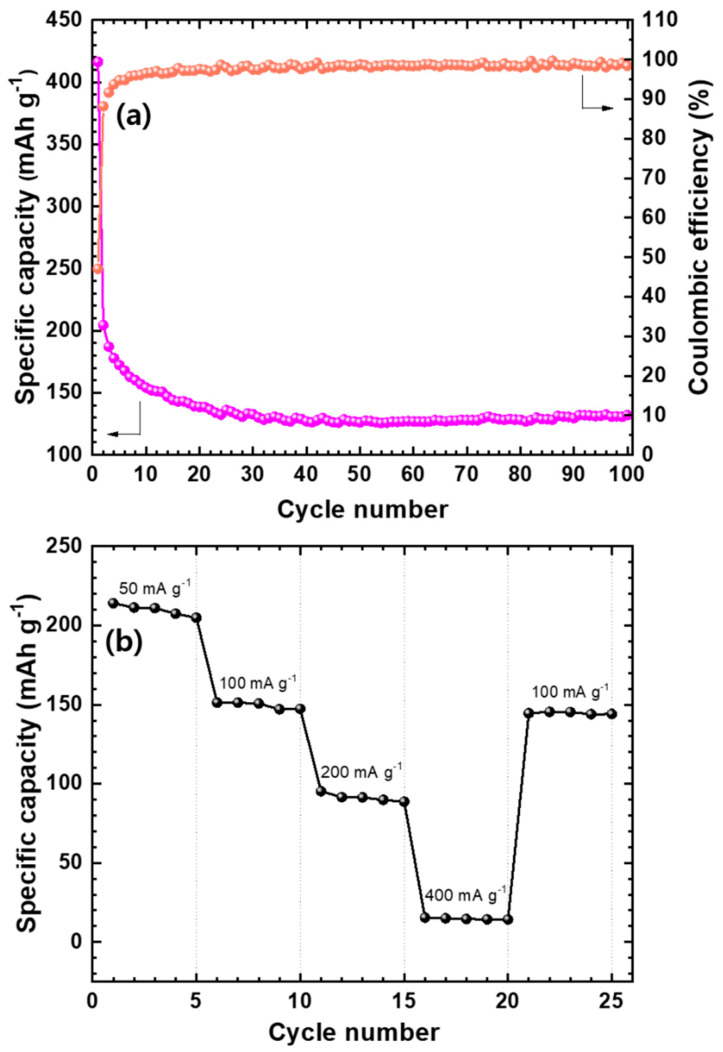
(**a**) Coulombic efficiency and battery cycling for Mo_2_C@CNT hybrid anode at 50 mA·g^−1^; (**b**) Rate capability profile at different current densities (50, 100, 200, and 400 mA·g^−1^) for the Mo_2_C@CNT hybrid anode.

The cycling behavior of Mo_2_C@CNT anode was performed and their results are provided in Figure 8a. Mo_2_C@CNT possesses high first discharge capacity (416 mAh g^−1^) and preserves 132 mAh g^−1^ capacity after 100 cycles due to their highly interacted synergistic reaction. Furthermore, the coulombic efficiency of the composite maintains at around 98%, which shows the stabile performance for LIBs. These enhanced characteristics are highly associated to the conducive (ionic and electronic) nature of the composite. Figure S4 shows the Nyquist lines after the 1st, 50th and 100th cycles for hybrid anode, which proves their stable conducting behavior. The rate performance of Mo_2_C@CNT anode was determined at diverse current densities as displayed in Figure 8b. The hybrid anode displays a capacity of approximately 211 mAh g^−1^ at 0.05 A g^−1^ and produces a 95 mAh g^−1^ capacity at 0.2 A g^−1^, which establishes its high rate behavior. Furthermore, the hybrid anode maintains its capacity at around 145 mAh g^−1^ when a current density is exchanged to 0.1 A g^−1^ from 0.4 A g^−1^. This illustrates the solidity and rate capability of the hybrid anode material, which is due to the synergistic effect between the CNT and Mo_2_C. The observed high stability is ascribed to the alleviation of Mo_2_C nanoparticle aggregation due to the inclusion of CNT, which presents the large pores because of the 3D structure formation and also provides a large area to contact with the Li^+^ electrolyte. Furthermore, the nanosized Mo_2_C particles increase the diffusion path of Li^+^ and the conductive nature of CNT connecting the nanoparticles, as well as offer fast electron transport and works as a buffer role to contain the volume expansion/contraction during charge/discharge.

## 4. Conclusions

In this work, the Mo_2_C@CNT hierarchical hybrid nanosheets were developed using a facile sonication process trailed by carbonization reaction and engaged as an anode electrode for LIBs. Specifically, the highly conductive CNT network acted as a template to inhibit the restacking/accretion of Mo_2_C particles and improved transport behavior in the hybrid material. The modified morphological characteristics were evidently proved by the FESEM and HRTEM results. The hierarchical Mo_2_C@CNT hybrid structures achieved a rescindable capacity of 132 mAh g^−1^ at 50 mA g^−1^ current density even after 100 cycles, showing an excellent cyclic life with admirable robustness due to the highly active interface engineering between the carbon materials. The enhanced storage kinetics of the Mo_2_@CNT hybrid structure create a viable and simple anode material preparation for effective LIBs electrochemical energy storage applications.

## Data Availability

The data presented in this study are available on request from the corresponding author.

## Supplementary Materials

The following are available online at https://www.mdpi.com/article/10.3390/nano11092195/s1, Figure S1: (a) Elemental mapping FESEM image of Mo_2_C@CNT hybrid and their elements distribution (b) Mo and (c) C, Figure S2: Survey XPS spectrum of Mo_2_C@CNT, Figure S3: Nyquist plots of the CNT, Mo_2_C, and Mo_2_C@CNT hybrid anodes, Figure S4: EIS plots for the Mo_2_C@CNT anode after 1st, 50th and 100th cycle.
