# Supplementary material for: Hierarchical Mo2C@CNT Hybrid Structure Formation for the Improved Lithium-Ion Battery Storage Performance
Source: Nanomaterials (Basel). 2021 Aug 26;11(9):2195. doi: 10.3390/nano11092195 (PMC8470648; doi:10.3390/nano11092195)
Supplement: Supplementary file 1 [file nanomaterials-11-02195-s001.zip › nanomaterials-1291679-supplementary.pdf]

Supplementary Information

Hierarchical Mo_2C@CNT Hybrid Structure Formation for the Improved Lithium-Ion Battery Storage Performance

Sajjad Hussain [1,2], Shoaib Muhammad [3], Muhammad Faizan [4,5], Kyung-Wan Nam [4], Hyun-Seok Kim [6], Dhanasekaran Vikraman [6,*] and Jongwan Jung [1,2,*]

[1] Hybrid Materials Center (HMC), Sejong University, Seoul 05006, Korea; shussainawan@gmail.com
[2] Department of Nanotechnology and Advanced Materials Engineering, Sejong University, Seoul 05006, Korea
[3] Department of Energy Science, Sungkyunkwan University, Suwon 16419, Korea; mshoaibce@gmail.com (S.M.)
[4] Department of Energy & Materials Engineering, Dongguk University-Seoul, Seoul 04620, Korea; faiz-ijaff@gmail.com (M.Z.); knam@dongguk.edu (K.-W.N.)
[5] Department of Materials Engineering, NED University of Engineering and Technology, Karachi, Pakistan
[6] Division of Electronics and Electrical Engineering, Dongguk University-Seoul, Seoul 04620, Korea; hyunseokk@dongguk.edu
* Correspondence: v.j.dhanasekaran@gmail.com (D.V.); jwjung@sejong.ac.kr (J.J.); Tel.: +82-2-3408-3688 (J.J.); Fax: +82-2-3408-4342 (J.J.).

Characterization Details

Field emission scanning electron microscopy (HITACHI S-4700, Tokyo, Japan) was used to characterize the morphological properties and elemental distributions. High resolution transmission electron microscopy (HRTEM, JEOL-2010F) with an operating voltage of 200 keV was used to analyze the atomic structures. The Raman spectroscopy (Renishaw inVia RE04, Gloucestershire, United Kingdom) measurements were made under ambient conditions using the 512 nm Ar laser source with a 1 μm laser spot size and 30 seconds scan speed. The structural properties were illustrated by Rigaku X-ray diffractometer (XRD) (Tokyo, Japan) with Cu-K_α radiation (0.154 nm) at 40 kV and 40 mA. For chemical composition and binding energy, the X-ray photoelectron spectroscopy (XPS) measurements were taken using an Ulvac PHI X-tool spectrometer (Kanagawa, Japan) with Al K_α X-ray radiation (1486.6 eV).

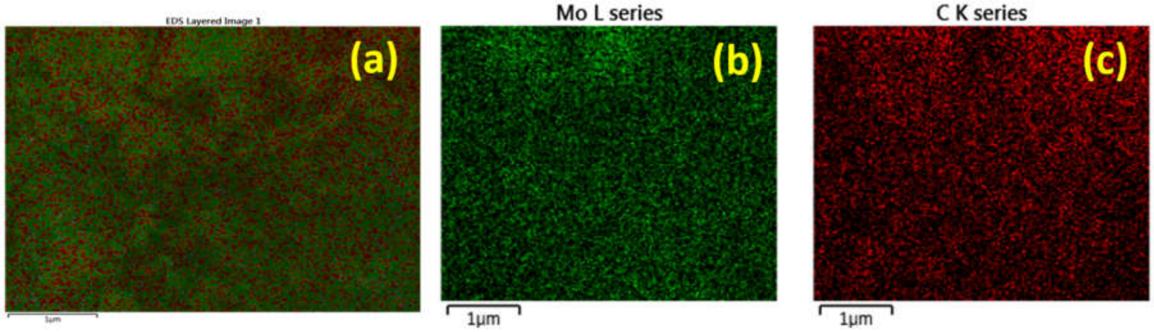

Figure S1. (**a**) Elemental mapping FESEM image of Mo_2C@CNT hybrid and their elements distribution (**b**) Mo and (**c**) C.

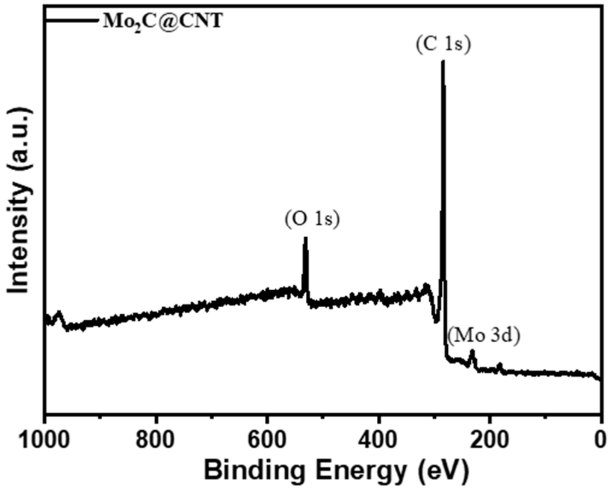

Figure S2. Survey XPS spectrum of Mo_2C@CNT.

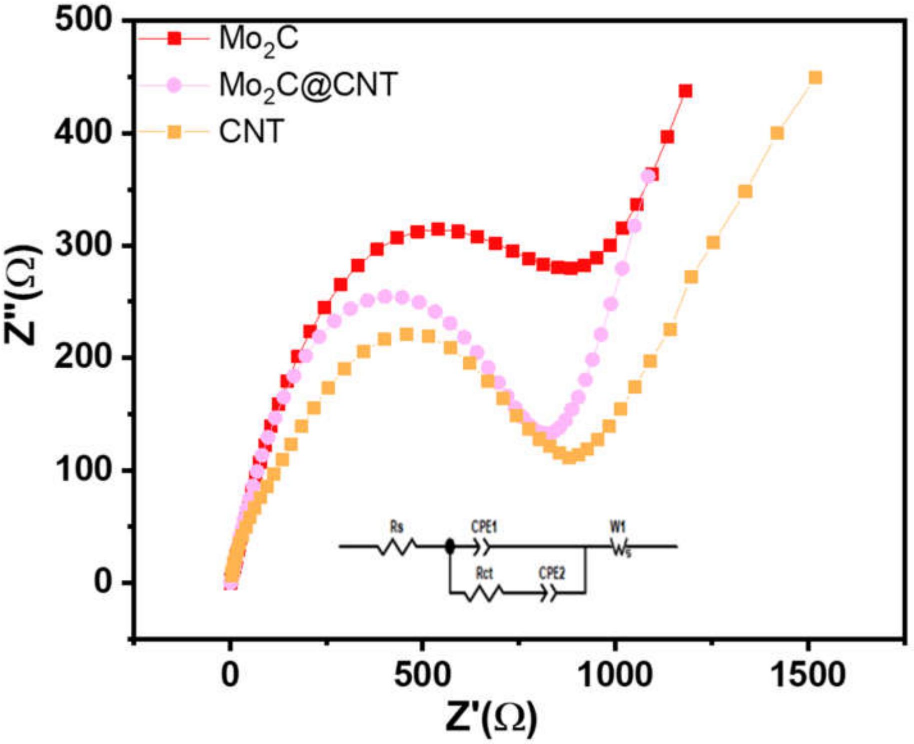

Figure S3. Nyquist plots of the CNT, Mo_2C, and Mo_2C@CNT hybrid anodes.

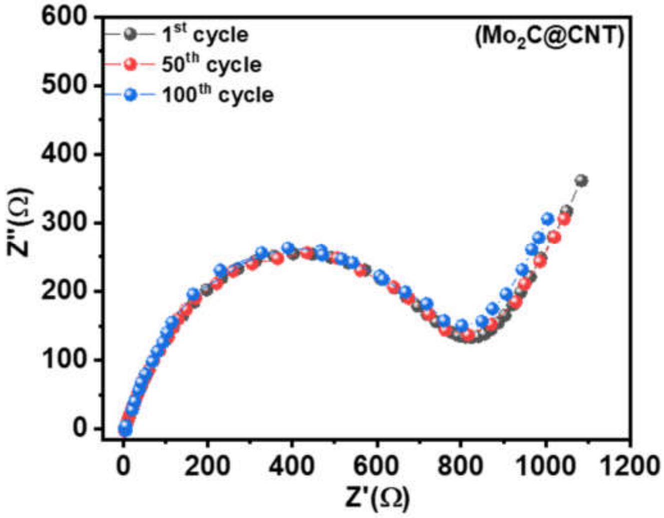

Figure S4. EIS plots for the Mo_2C@CNT anode after 1st, 50th and 100th cycle.
